# The fear of cancer recurrence and progression in patients with pancreatic cancer

**DOI:** 10.1007/s00520-022-06887-w

**Published:** 2022-02-15

**Authors:** Esther N. Pijnappel, Willemieke P. M. Dijksterhuis, Mirjam A. G. Sprangers, Simone Augustinus, Judith de Vos-Geelen, Ignace H. J. T. de Hingh, Izaak Q. Molenaar, Olivier R. Busch, Marc G. Besselink, Johanna W. Wilmink, Hanneke W. M. van Laarhoven, for the Dutch Pancreatic Cancer Group

**Affiliations:** 1 Department of Medical Oncology, Amsterdam UMC, University of Amsterdam, Cancer Center Amsterdam, Meibergdreef 9, 1105 AZ Amsterdam, The Netherlands; 2 Netherlands Cancer Registry, Netherlands Comprehensive Cancer Organization (IKNL), PO Box 19079, Utrecht, 3501 DB The Netherlands; 3 Department of Psychology, Amsterdam UMC, University of Amsterdam, Amsterdam, The Netherlands; 4 Department of Surgery, Amsterdam UMC, University of Amsterdam, Cancer Center Amsterdam, Amsterdam, The Netherlands; 5 Department of Internal Medicine, Division of Medical Oncology, GROW–School for Oncology and Developmental Biology, Maastricht UMC+, P. Debyelaan 25, Maastricht, 6229 HX The Netherlands; 6 Department of Surgery, Catharina Hospital, Eindhoven, The Netherlands; 7 Department of Surgery, University Medical Centre Utrecht, Utrecht, The Netherlands

**Keywords:** Pancreatic neoplasms, Pancreatic ductal adenocarcinoma, Fear of cancer recurrence, Fear of cancer progression

## Abstract

**Purpose:**

It is plausible that patients with pancreatic cancer experience fear of tumor recurrence or progression (FOP). The aim of this study was to compare FOP in patients with pancreatic cancer treated with surgical resection, palliative systemic treatment, or best supportive care (BSC) and analyze the association between quality of life (QoL) and FOP and the effect of FOP on overall survival (OS).

**Methods:**

This study included patients diagnosed with pancreatic cancer between 2015 and 2018, who participated in the Dutch Pancreatic Cancer Project (PACAP). The association between QoL and WOPS was assessed with logistic regression analyses. OS was evaluated using Kaplan–Meier curves with the log-rank tests and multivariable Cox proportional hazard analyses adjusted for clinical covariates and QoL.

**Results:**

Of 315 included patients, 111 patients underwent surgical resection, 138 received palliative systemic treatment, and 66 received BSC. Patients who underwent surgical resection had significantly lower WOPS scores (i.e., less FOP) at initial diagnosis compared to patients who received palliative systemic treatment or BSC only (*P* < 0.001). Better QoL was independently associated with the probability of having a low FOP in the BSC (OR 0.95, 95% CI 0.91–0.98) but not in the surgical resection (OR 0.97, 95% CI 0.94–1.01) and palliative systemic treatment groups (OR 0.97, 95% CI 0.94–1.00). The baseline WOPS score was not independently associated with OS in any of the subgroups.

**Conclusion:**

Given the distress that FOP evokes, FOP should be explicitly addressed by health care providers when guiding pancreatic cancer patients through their treatment trajectory, especially those receiving palliative treatment or BSC.

**Supplementary Information:**

The online version contains supplementary material available at 10.1007/s00520-022-06887-w.

## Introduction

Pancreatic ductal adenocarcinoma (PDAC) is a lethal condition with 80–85% of the newly diagnosed patients suffering from locally advanced or metastatic disease. Despite advances in treatment, PDAC is still characterized by a very poor prognosis with a 5-year survival of 3.5% [1]. Due to late detection and its unfavorable prognosis, even when treatment is started, the risk of progression or recurrence, eventually leading to death, is high.

Fear of progression (FOP) is defined as “patients’ fear that the illness will progress or that it will recur” and is one of the most frequent distress symptoms of patients with cancer [2, 3]. There is a growing tendency to approach FOP as a multidimensional concept; a combination of cognitive, behavioral, and emotional concerns of cancer patients [4, 5]. Research has shown that about 50% of cancer patients experience a moderate to a high degree of FOP of the disease [2, 6, 7]. High FOP prevalence was reported in 56% of patients with first-ever cancer diagnosis [2, 8]. In cancer survivors, FOP is also high; 24–70% in breast cancer, 35% in head and neck cancer, and 31% in testicular cancer survivors [9–13]. The prevalence of FOP in pancreatic cancer patients is unknown.

Recent studies identified potential factors that were found to correlate with and predict FOP. Increasing age, a higher disease stage, a higher number of somatic symptoms, and impaired quality of life (QoL) were found to be correlated with higher FOP [14, 15]. All of these variables are also predictive of a higher chance of imminent death. We assume that the treatment intent (curative versus palliative) in pancreatic cancer patients may affect FOP. Therefore patients are categorized based on their therapy (surgical resection, palliative systemic treatment, and best supportive care [BSC]). We also hypothesize that FOP might decrease over time in the individual patients undergoing curative treatment and may increase in patients receiving palliative treatment; therefore, it is important to examine FOP over time.

To the best of our knowledge, no data are available on the relationship between these correlating variables and FOP in pancreatic cancer patients. In the context of pancreatic cancer, such a relationship is of particular interest, given its poor prognosis, high symptom burden, and relatively poor QoL [16]. Specifically, the question arises whether disease stage, symptom burden, and QoL are also discriminative for different levels of FOP [17]. Hence, in this study, we will examine the association between overall QoL (measured with a summary score including in particular symptoms) and FOP.

Previously, an association has been reported between an increased level of FOP and inferior overall survival (OS) in lymphoma patients [18]. It might be hypothesized that this relationship is indirect, where patients with elevated levels of FOP experience a higher number of symptoms related to a higher tumor load and therefore have lower chances of survival [19, 20]. We will investigate the hypothesis that FOP is associated with OS.

Therefore, the aims of this study are to compare the prevalence of FOP, analyze changes of FOP over time, and examine the association between QoL and FOP and the association between FOP and OS.

## Materials and methods

### Data collection

All patients diagnosed with pancreatic ductal adenocarcinoma, excluding patients with neuroendocrine tumors, between 2015 and 2018 who participated in the Pancreatic Cancer Project (PACAP) were selected from the Netherlands Cancer Registry (NCR). The NCR is a population-based registry and is linked to the pathological reports of all histologically proved cancer diagnoses in the Netherlands. The NCR comprises data of more than 17 million (also deceased) individuals of the Dutch population and contains a fair representation of all the pancreatic cancer patients nationally. PACAP is a nationwide Dutch project that was founded in 2013 by the Dutch Pancreatic Cancer Group (DPCG) and comprises data on clinical information and patient-reported outcome measures (PROMs) [21, 22]. Information on patients (gender, age, World Health Organization (WHO) performance status, number of comorbidities), tumor (location, stage), treatment (surgical resection, systemic treatment, BSC), and day to the last follow-up were identified from the NCR and were matched with the PROMS data for analyses.

Patients were categorized based on their initial treatment: surgical resection, palliative systemic treatment, or BSC. This study was designed in accordance with the Strengthening the Reporting of Observational Studies in Epidemiology (STROBE) guidelines [23].

### Patient-reported outcome measures

FOP was assessed with the Worry of Progression Scale (WOPS), which is part of the PACAP survey. The WOPS questionnaire is a modified Dutch seven-item version of the six-item English Cancer Worry Scale (CWS), enquiring about the fear of cancer progression and the impact of fear on daily functioning [24, 25]. In the WOPS questionnaire, we used the six questions of the CWS and adapted these to also include fear of progression, instead of fear of recurrence only [25, 26]. We added one question about the fear of having no medical treatment options left (see Supplementary Information). A four-point Likert scale was used to rate the seven items ranging from 1 (“never” or “not at all”) to 4 (“almost always” or “very much”). The sum score ranged from 7 to 28, with higher scores indicating more fear. A WOPS score below the median (i.e., < 15) was defined as low.

Cancer-specific health-related quality of life (HRQL) was assessed with the European Organization for Research and Treatment of Cancer (EORTC) Core Quality of Life Questionnaire (QLQ-C30) [27, 28]. Its 30 questions are combined to form five multi-item functioning scales on physical, role, social, emotional, and cognitive functioning; three multi-item symptom scales on fatigue, nausea, vomiting, and pain; six single-item symptoms scores on dyspnea, insomnia, appetite loss, constipation, diarrhea, and financial impact; and a two-item global quality of life scale [28]. QLQ-C30 was rated on a four-point Likert scale ranging from “not at all” to “very much,” except for the two questions on global QoL that employed a seven-point Likert scale, ranging from “not at all” to “very much.” The original score was linearly transformed into scores ranging between zero and 100. As a measure of overall QoL, we used the summary score, which is defined as the mean of the combined QLQ-C30 scores excluding global QoL and financial impact questions [29, 30]. A higher summary score indicates a better overall QoL.

These PROMs were administered at baseline and 3, 6, 9, 12, 18, 24, and 36 months after baseline and yearly until death or study withdrawal. The WOPS and QLQ-C30 data obtained at baseline and 3 and 9 months after baseline were used for the current analyses. For the WOPS to be defined as a baseline measure, it had to be completed at any point in time after diagnosis (best supportive care), filled out before surgical resection (between diagnosis and surgical resection), or before the start of palliative systemic treatment or within 7 days after the start of palliative systemic treatment (since it is not likely that one cycle of chemotherapy will affect WOPS scores). Only patients with a baseline questionnaire were included in the analyses.

### Statistical analysis

Data were analyzed with SAS software (version 9.4, SAS Institute, Cary, NC, USA). Baseline characteristics were presented with means and standard deviations (SD) for continuous variables or medians and interquartile ranges (IQRs) for categorical variables. The latter variables were described with absolute numbers and percentages. Differences in patient and tumor characteristics among the treatment groups (surgical resection, palliative systemic treatment, and BSC) were tested with chi-square tests combined with Fisher’s exact tests where suitable. The difference in mean WOPS score between the three treatment groups was tested with ANOVA. The proportion of high versus low WOPS scores between the three treatment groups was tested with chi-square tests. The association between QoL and WOPS scores was assessed with logistic regression analysis adjusted for gender, age, comorbidity, performance status and year of diagnosis in all subgroups, and a number of metastatic locations in the palliative systemic treatment and BSC groups. OS was defined as the time interval from diagnosis until the end of follow-up or death, updated on February 1, 2020. We calculated OS from the day of diagnosis and not from the day of completion of the questionnaires because all other patient and tumor characteristics were defined on the day of diagnosis as well. Kaplan–Meier analyses with log-rank test were used to examine median OS for each treatment group (surgical resection, palliative systemic treatment, BSC) and each group according to their WOPS score (high vs low). With multivariable Cox proportional hazard regression analyses, the independent association between WOPS scores at baseline and OS was identified, adjusted for age, gender, the number of comorbidities, performance status, year of diagnosis and QoL (in all subgroups), and the number of metastatic organ sites (in the systemic treatment and BSC groups). The probability of a type-I error was set at 0.05 without correction for multiple testing since we only compared three treatment groups.

## Results

### Patient characteristics

**Fig. 1:**
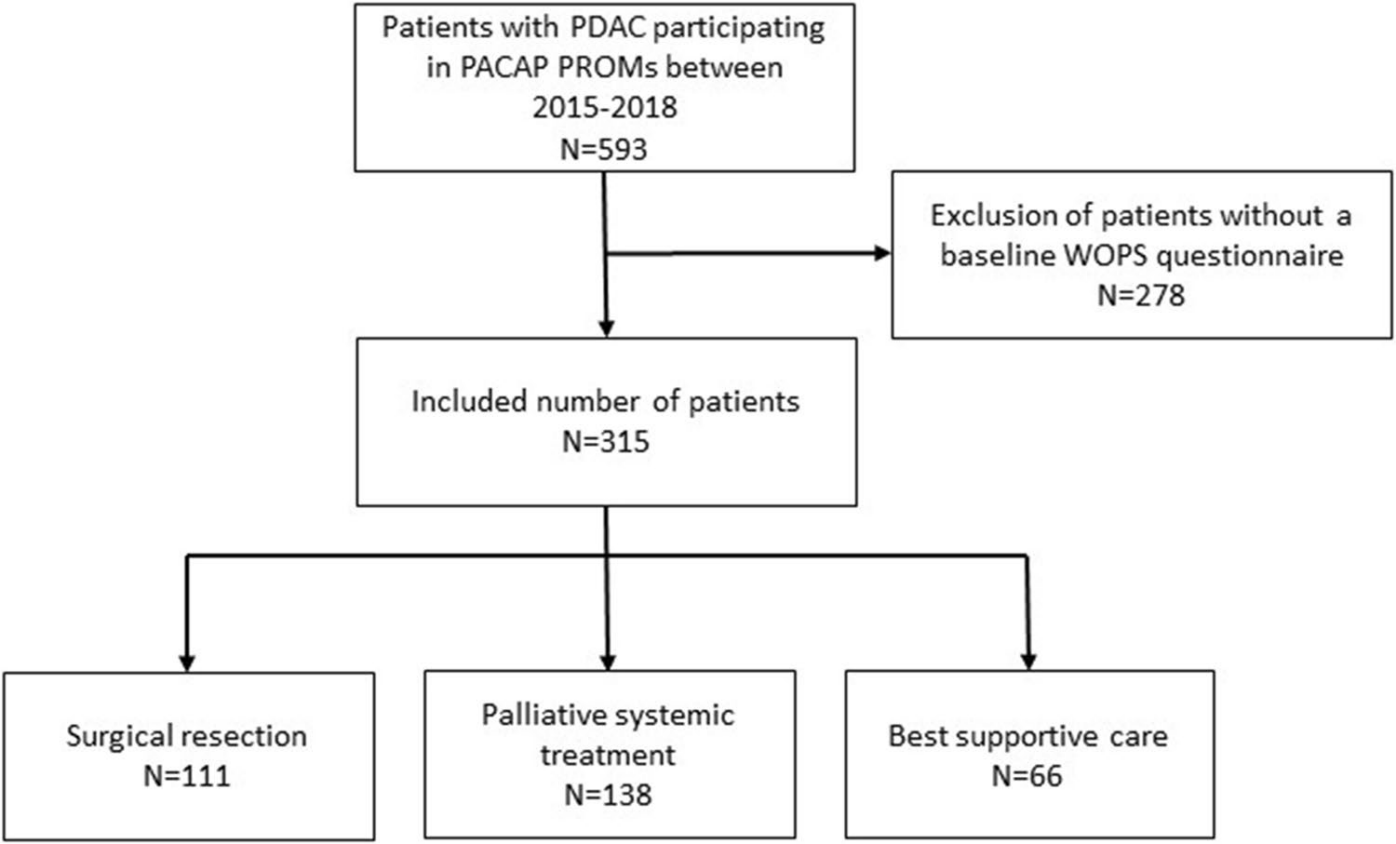
Flow diagram of patient inclusion. Abbreviation: PDAC, pancreatic ductal adenocarcinoma; PACAP, Pancreatic Cancer Project; PROMs, patient-reported outcome measures; WOPS, Worry of Progression Scale; *N*, number

A total of 593 patients with PDAC participated in the PACAP cohort between 2015 and 2018, 278 of whom were excluded as they lacked a baseline WOPS questionnaire (Fig. 1). The majority of the remaining 315 patients was male (55%) with a median age of 66 years (IQR 60–72; Table 1). Most patients had pancreatic head tumors (60%). No comorbidities (42%) and a performance status of 0 or 1 was observed in the majority of patients (70%). Of all 315 included patients, 111 (35%) underwent surgical resection, 138 (44%) received palliative systemic treatment, and 66 (21%) received BSC (Table 1). After 3 months, 193 patients and after 9 months 95 patients completed the WOPS questionnaires (Supplementary Table 1).

**Table 1:** Baseline characteristics

Variable	Total (*n* = 315)	Surgical resection (*n* = 111)	Palliative systemic treatment (*n* = 138)	Best supportive care (*n* = 66)
Gender, *n* (%)				
Male Female	174 (55%) 141 (45%)	68 (61%) 43 (39%)	72 (52%) 66 (48%)	34 (52%) 32 (48%)
Age years, median (IQR)	66 (60–72)	66 (61–72)	65 (57–70)	71 (65–76)
< 55 55–64 65–74 ≥ 75	41 (14%) 86 (27%) 140 (44%) 48 (15%)	15 (13%) 33 (30%) 50 (45%) 13 (12%)	23 (17%) 41 (30%) 62 (45%) 12 (8%)	3 (5%) 12 (18%) 28 (42%) 23 (35%)
Tumor location, *n* (%				
Head Body Tail Overlapping sites Pancreas NOS	191 (60%) 50 (16%) 43 (14%) 21 (7%) 10 (3%)	90 (80%) 3 (3%) 14 (13%) 1 (1%) 3 (3%)	65 (47%) 35 (25%) 20 (15%) 14 (10%) 4 (3%)	36 (55%) 12 (18%) 9 (14%) 6 (9%) 3 (4%)
Number of comorbidities, *n* (%)				
0 1 2 Missing	131 (42%) 93 (29%) 48 (15%) 43 (14%)	39 (35%) 37 (33%) 15 (14%) 20 (18%)	61 (44%) 37 (27%) 22 (16%) 18 (13%)	31 (47%) 19 (29%) 11 (17%) 5 (7%)
Performance status, *n* (%)				
WHO 0–1 WHO 2 WHO 3–4 Unknown	221 (70%) 25 (8%) 10 (3%) 59 (19%)	77 (69%) 4 (4%) 2 (2%) 28 (25%)	112 (81%) 10 (7%) 1 (1%) 15 (11%)	32 (48%) 11 (17%) 7 (11%) 16 (24%)
Year of diagnosis, *n* (%)				
2015 2016 2017 2018	36 (11%) 33 (10%) 121 (38%) 125 (41%)	13 (12%) 14 (13%) 39 (35%) 45 (40%)	14 (10%) 17 (12%) 54 (39%) 53 (39%)	9 (14%) 2 (3%) 28 (42%) 27 (41%)
Number of metastatic sites, *n* (%)				
0 1 ≥ 2	203 (64%) 75 (24%) 37 (12%)	111 (100%) 0 (0%) 0 (0%)	65 (47%) 47 (34%) 26 (19%)	29 (44%) 26 (39%) 11 (17%)

Abbreviations: *n*, number; *IQR*, interquartile range; *NOS*, not other specified; *WHO*, World Health Organization

### Prevalence of WOPS scores over time

**Fig. 2:**
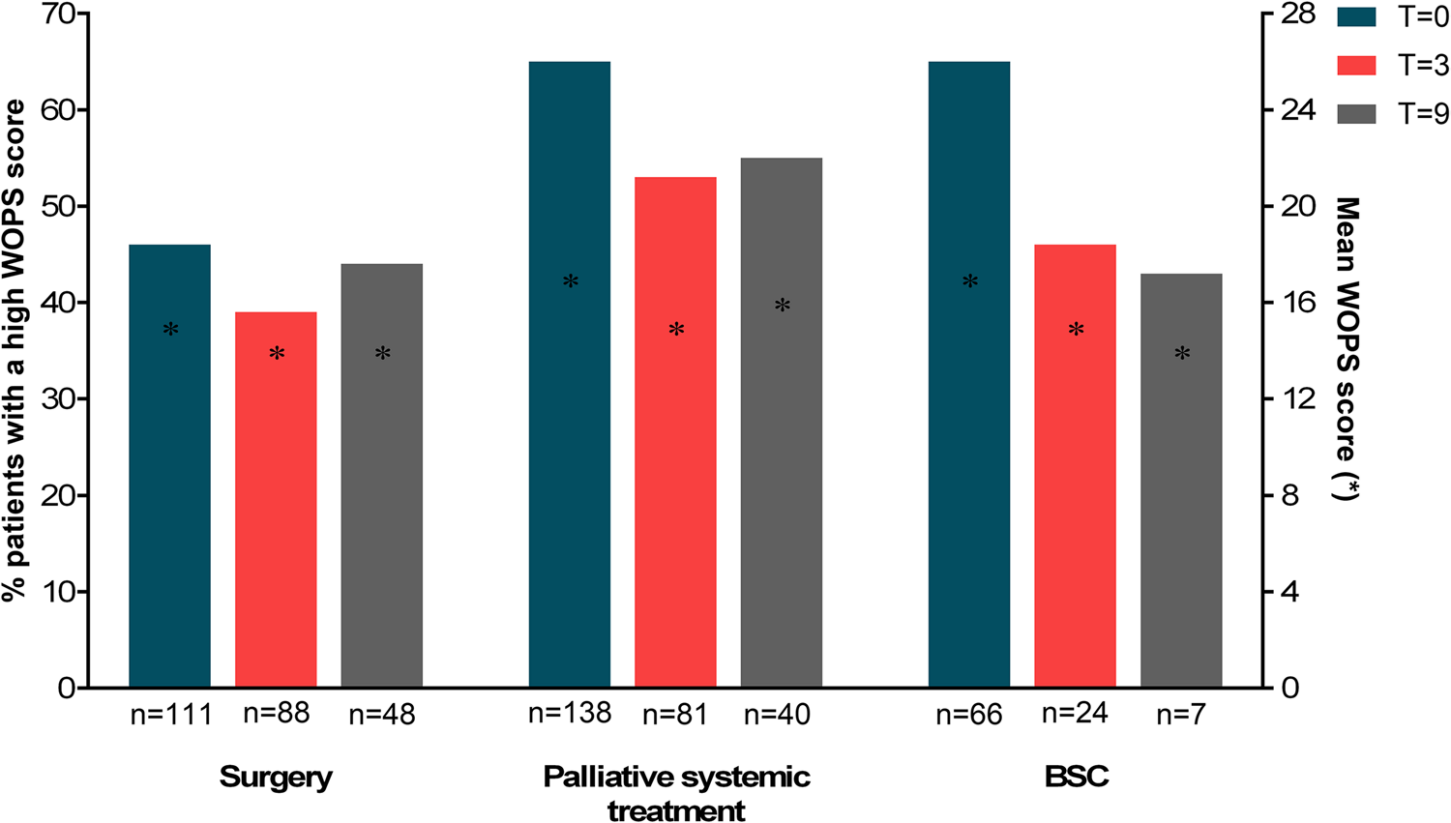
Percentage of patients with high WOPS scores over time for the different treatment groups (surgical resection, palliative systemic treatment, and BSC). Abbreviation: BSC, best supportive care; WOPS, Worry of Progression Scale; *T* = 0, baseline; *T* = 3, after 3 months; *T* = 9, after 9 months, *n*, number

At baseline, the mean WOPS score for all patients was 16 (SD 5), with 58% of the patients scoring above the median of 15 (i.e., high WOPS scores). The mean WOPS scores were 15 (SD 5), 17 (SD 5), and 17 (SD 6) for the surgical resection (*n* = 111), the palliative systemic treatment (*n* = 138), and BSC group (*n* = 66), respectively (Table 1). Patients who underwent surgical resection had significantly lower (mean) WOPS scores compared to patients in the palliative systemic treatment and BSC groups at baseline (*p* = 0.001 and *p* = 0.004; Supplementary Table 1, Fig. 2). WOPS scores at 3 months and 9 months did not differ across the subgroups (Supplementary Table 1).

### Relationship of QoL with WOPS

Only for the BSC group, a better QoL score was independently associated with the probability of having a low FOP (OR 0.94; 95% CI 0.91–0.98) (Supplementary Table 2). For the surgical resection and palliative systemic treatment groups, higher QoL was not statistically significantly associated with lower FOP (OR 0.97; 95% CI 0.94–1.01 and OR 0.97; 95% CI 0.94–1.00).

### Survival and FOP

**Fig. 3:**
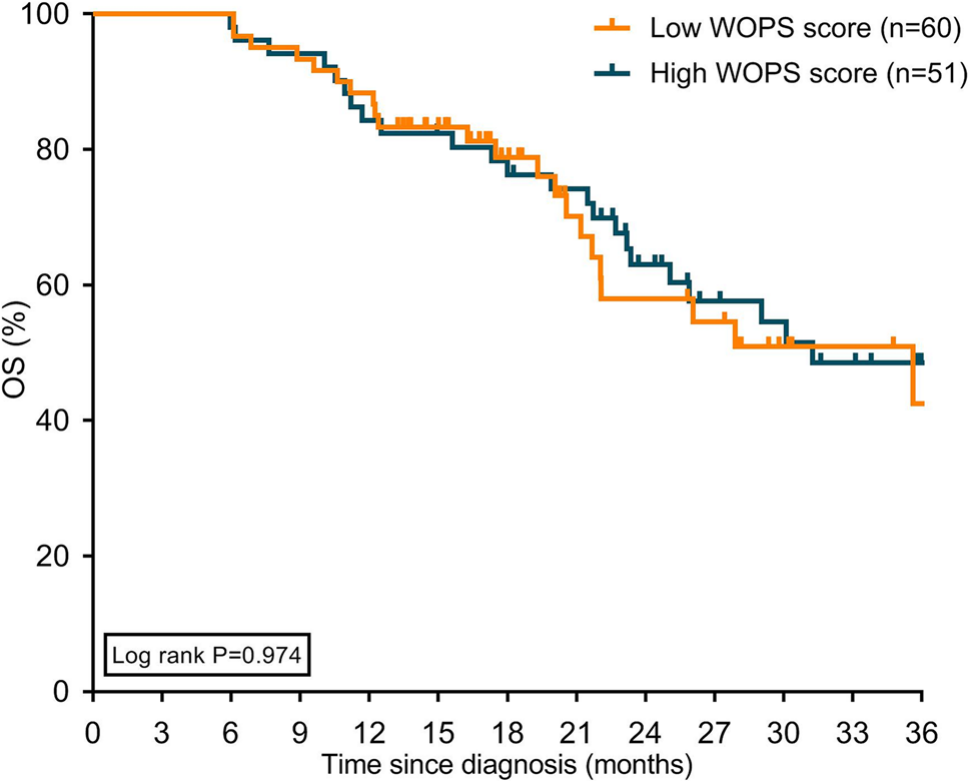
Kaplan–Meier curves displaying overall survival in patients with high and low WOPS scores treated with surgical resection. Abbreviation: WOPS, Worry of Progression Scale; OS, overall survival

**Fig. 4:**
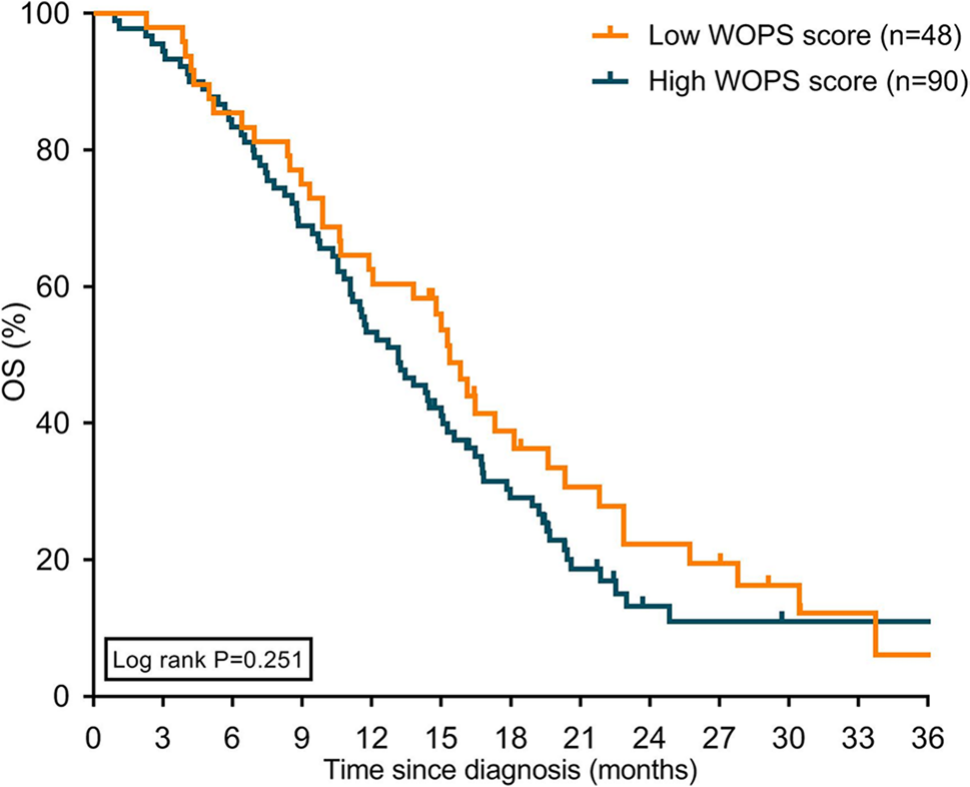
Kaplan–Meier curves displaying overall survival in patients with high and low WOPS scores treated with palliative systemic treatment. Abbreviation: WOPS, Worry of Progression Scale; OS, overall survival

**Fig. 5:**
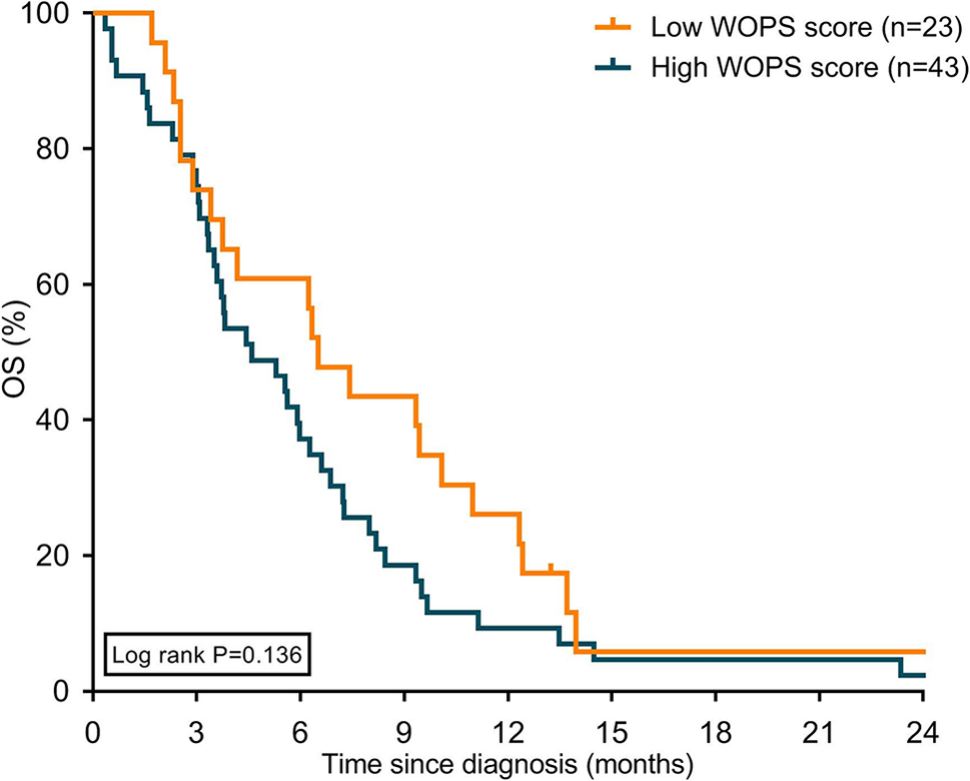
Kaplan–Meier curves displaying overall survival in patients with high and low WOPS scores treated with BSC. Abbreviation: WOPS, Worry of Progression Scale; OS, overall survival

Median OS was 31.2 months for patients in the surgical resection group, 14.1 months for patients undergoing palliative systemic treatment, and 5.6 months for patients who received BSC (Supplementary Fig. 1). Median OS did not statistically significantly differ between patients with a high or low WOPS score for all treatment subgroups (Fig. 3, 4, 5).

WOPS scores were not independently associated with OS in all treatment subgroups after adjustment for patient and tumor characteristics (Supplementary Table 3).

## Discussion

To the best of our knowledge, this is the first study assessing FOP in patients with pancreatic cancer. As expected, patients who underwent surgical resection had significantly lower baseline WOPS scores compared to patients in the palliative systemic treatment and BSC group. Better QoL was only independently associated with the probability of having a low FOP in the BSC group. A high WOPS score at baseline was not independently associated with OS after adjustment for patient and tumor characteristics for any of the treatment subgroups.

Although previous studies, describing other cancer types than pancreatic cancer, suggested that disease- and treatment-related factors may be less relevant to FOP [2, 15], in our study, patients who received palliative systemic treatment or BSC presented more often with high WOPS scores at baseline, reflecting more fear compared to patients who underwent surgical resection. This trend was only observed at baseline, not at the other time points as we expected. This result may be explained by the poor overall survival of PDAC patients, especially in the advanced disease setting. The median overall survival of patients with PDAC treated with palliative chemotherapy or best supportive care is 6 months and 1.5 months, respectively [1, 31]. Indeed, surgery is the treatment of choice for patients with a limited disease without metastases and provides the best chance for long-term survival without disease recurrence [1]. Unfortunately, surgery is not an option for patients with advanced, metastatic disease. This could explain why patients who are planning to undergo curative surgery have less FOP compared to patients receiving palliative treatment. Other prognostic studies also reported elevated levels of FOP because of worsening of the prognosis due to an advanced disease stage [8, 32, 33]. In addition, studies have shown that patients’ expectations are often too high for cancer surgery in general [34–36].

WOPS scores in our study remained stable and did not increase over time in all subgroups. This is in line with the outcomes of other studies that showed a slight reduction in FOP in the first months after baseline score and stabilization afterward or that showed a steady and significant decline after diagnosis [8, 37]. Higher scores at baseline might be explained by the fact that FOP is related to the elevated overall psychological distress at diagnosis [37]. The statistical phenomenon “regression to the mean” may also explain, in part, the decrease of FOP following baseline [8].

This study showed that better QoL was statistically significantly associated with the probability of having a low FOP in patients who received BSC (OR 0.94). The same trend was found in patients who were treated with surgical resection or palliative systemic treatment (both with an OR of 0.97), although these ORs were not statistically significant. These results are in line with other studies showing increasing or maintaining QoL may reduce fear [38–40]. Acceptance and recognition of a patients’ FOP should be an important treatment goal in patients with PDAC. The fact that cancer brings a risk to psychological wellbeing should be a subject of discussion in the consultation room to determine the needs of a patient in order to find the most suitable psychological support [41]. A medical provider has a signaling function and should refer patients to a mental health professional if necessary. However, research on supportive care or psychological support, specifically for patients with PDAC, is limited. Studies among patients with other cancer types suggest that study nurses who coach the patients during their entire treatment process, optimize information provision, and provide supportive care were found to have a beneficial effect on psychosocial functioning and acceptance of the disease. These studies also identified a role of peer support groups showing a favorable outcome on QoL [42–44]. Other studies have shown that psychological interventions performed by a mental health professional help to reduce feelings of distress for patients with other cancer types than PDAC and are a necessary element of comprehensive cancer care [45, 46]. Further research on this topic is essential in order to identify the supportive care and psychological assistance for this patient population.

Currently, there is only one study in cancer that found that severe FOP in lymphoma patients was associated with an increased mortality risk [18]. In our study, we did not observe a significant association between FOP and survival. However, despite being not significant, the numerically higher median OS observed in patients with low WOPS scores compared to high WOPS scores in the palliative systemic treatment and BSC groups tend toward an association, indicating that WOPS scores are related to survival. Possibly, the subgroups were too small to reach statistically significant associations. More data are needed to draw conclusions on the prognostic effect of FOP on OS.

A limitation of this study is that 53% of the patients had to be excluded from the analysis because the baseline WOPS was not completed and that a considerable part of the included patients did not complete questionnaires at 3 or 9 months, which limited the group sizes. Second, there might be a selection bias in the data collection of the PACAP PROMs. In our study, 35% of the patients received surgical resection, and 44% was treated with palliative systemic therapy. These percentages are higher compared to the average in The Netherlands, with a resection rate of around 15% and 25% of the patients receiving palliative chemotherapy [47]. In addition, patients in both treatment groups show better OS compared to other real-world studies [47–49], suggesting that patients with a better condition more often completed these PROMs. Fourth, if patients filled out their baseline questionnaires before a decision about a specific treatment was made, this may have led to uncertainty and could also be an explanation for the high FOP levels at diagnosis. As a result, the FOP levels decrease after 3 months because this second time point would fall after the start of treatment.

In conclusion, this real-world study is the first to provide information about the FOP in patients with PDAC. We observed that patients with PDAC report FOP at diagnosis, which stabilized over time. Patients who received palliative treatment or BSC had a higher FOP compared to surgically treated patients at baseline. Better QoL was associated with the probability of having a low FOP in patients receiving BCS. Given the distress that FOP evokes, FOP should be explicitly addressed by health care providers when guiding pancreatic cancer patients through their treatment trajectory.

## Supplementary Information

Below is the link to the electronic supplementary material.

Supplementary file1 (DOCX 24 KB)

## Data Availability

The data underlying this article will be shared at reasonable request to the corresponding author.
