# Supplementary material for: The fear of cancer recurrence and progression in patients with pancreatic cancer
Source: Support Care Cancer. 2022 Feb 15;30(6):4879–87. doi: 10.1007/s00520-022-06887-w (PMC9046341; doi:10.1007/s00520-022-06887-w)
Supplement: Supplementary file 1 — Supplementary file1 (DOCX 24 KB) [file 520_2022_6887_MOESM1_ESM.docx]

Supplementary Material

**Supplementary material Methods**

**CWS 6-items (original English version)**

1. How often have you thought about your chances of getting cancer (again)?
2. Have these thoughts affected your mood?
3. Have these thoughts interfered with your ability to do daily activities?
4. How concerned are you about the possibility of getting cancer (again) one day?
5. How often do you worry about developing cancer (again)?
6. How much of a problem is this worry?

**CWS 8-items including the two added questions (validated)**

1. How often have you thought about your chances of getting cancer (again)?
2. Have these thoughts affected your mood?
3. Have these thoughts interfered with your ability to do daily activities?
4. How concerned are you about the possibility of getting cancer (again) one day?
5. How often do you worry about developing cancer (again)?
6. How much of a problem is this worry?
7. **How often do you worry about the chance of family members developing cancer?**
8. **How concerned are you about the possibility that you will ever need surgery (again)?**

**WOPS questionnaire including fear of recurrence and progression and one extra question in addition to the CWS 6-items, as used in the PACAP questionnaire**

1. How often have you thought about your chances of getting cancer again or cancer progression?
2. How often have these thoughts affected your mood?
3. How often have these thoughts about recurrence of progression interfered with your ability to do daily activities?
4. How concerned are you about the possibility of getting cancer again or cancer progression one day?
5. How often do you worry about developing cancer again or cancer progression?
6. How much of a problem is this worry?
7. **How concerned are you about the possibility that you will have no more medical treatment options?**

**Supplementary table 1. WOPS scores over time for the different treatment groups**

| **WOPS score** | **Surgical resection** | **Palliative systemic treatment** | **Best supportive care** | **p-value** |
| --- | --- | --- | --- | --- |
| **baseline**  Mean score (SD)  Score high*  Score low | N=111  15 (5)  51 (46%)  60 (54%) | N=138  17 (5)  90 (65%)  48 (35%) | N=66  17 (6)  43 (65%)  23 (35%) | 0.001 ^a^  0.004^b^ |
| **3 months**  Mean score(SD)  High*  Low | N=88  14 (5)  34 (31%)  54 (49%) | N=81  15 (4)  43 (31%)  38 (28%) | N=24  15 (6)  11 (16%)  13 (20%) | 0.253 ^a^  0.169^b^ |
| **9 months**  Mean score (SD)  High*  Low | N=48  14 (5)  21 (19%)  27 (24%) | N=40  16 (5)  22 (16%)  18 (13%) | N=7  14 (5)  3 (5%)  4 (6%) | 0.226 ^a^  0.549^b^ |

*Abbreviation: WOPS=* *Worry of Progression Scale, SD=standard deviation***,** *^a^ = ANOVA tests for the comparison of the continues variable WOPS score between the surgical resection, palliative treatment and best supportive care groups; ^b^ = Chi squared tests of the comparison of high and low scores across the surgical resection, palliative systemic treatment and best supportive care groups.*

** A high WOPS score is defined as a WOPS score >15 (i.e. above median)*

**Supplementary table 2. Multivariable logistic regression Quality of life and WOPS score (high/low)**

Adjusted for: gender, age, number of comorbidities, performance status, year of diagnosis in all subgroups, and number of metastases locations in the palliative systemic treatment and BSC groups.

|  | **Surgical resection** | **Palliative systemic treatment** | **Best supportive care** |
| --- | --- | --- | --- |
|  | **OR 95% CI P value** | **OR 95% CI P value** | **OR 95% CI P value** |
| **Quality of life** | 0.97 0.94-1.01 0.0979 | 0.97 0.94-1.00 0.069 | 0.94 0.91-0.98 **0.00320* |
| **Gender**  **Male**  **Female** | Ref  1.34 0.51-3.56 0.556 | Ref  3.08 1.33-7.14 **0.0087* | Ref  0.67 0.19-2.37 0.535 |
| **Age years**  **<55**  **55-64**  **65-74**  **≥75** | Ref  0.95 0.21-4.25 0.945  0.73 0.16-3.31 0.683  1.00 0.14-7.08 0.999 | Ref  0.62 0.19-2.01 0.427  1.22 0.40-3.70 0.726  1.75 0.28-10.96 0.549 | Ref  1.67 0.069-40.21 0.752  0.79 0.037-16.97 0.882  1.77 0.073-42.91 0.726 |
| **Performance status**  **WHO 0-1**  **WHO 2-4**  **Unknown** | Ref  0.38 0.036-3.94 0.839  0.13 0.024-0.63 **0.0186* | Ref  2.48 0.55-11.16 0.235  2.39 0.66-8.73 0.186 | Ref  1.09 0.23-5.16 0.910  0.90 0.18-4.49 0.895 |
| **Number of comorbidities**  **0**  **1**  **≥2**  **Unknown** | Ref  1.26 0.36-4.49 0.719  4.72 0.87-25.51 0.072  2.17 0.54-8.74 0.275 | Ref  2.60 0.91-7.43 0.0739  0.81 0.26-2.54 0.713  1.61 0.44-5.83 0.471 | Ref  0.70 0.14-3.50 0.667  0.22 0.031-1.61 0.137  0.42 0.029-6.11 0.524 |
| **Number of metastatic sites**  **0**  **1**  **2 or more** | NA | Ref  2.74 1.11-6.77 **0.0295*  1.24 0.42-3.71 0.701 | Ref  6.90 1.28-37.28 **0.0249*  4.90 0.61-39.64 0.137 |
| **Year of diagnosis**  **2015-2016**  **2017-2018** | Ref  0.55 0.20-1.55 0.258 | Ref  1.04 0.41-2.65 0.939 | Ref  0.21 0.034-1.37 0.103 |

*Abbreviations: WHO=World Health Organization, NA=not applicable, OR=odds ratio, 95% CI=95% confidence interval, Ref=reference group,
* statistically significant.*

**Supplementary table 3. Multivariable Cox-regression WOPS score and overall survival**

Multivariable Cox-regression analysis was stratified for the three treatment groups (surgical resection, palliative systemic treatment and BSC)
Adjusted for: gender, age, number of comorbidities, performance status, year of diagnosis and QoL in all treatment groups and only in the palliative systemic and BSC group adjusted for number of metastatic locations

Reference group: Low WOPS score

| **Parameter** | **Hazard ratio** | **95% Hazard Ratio**  **Confidence Limits** | | **P-value** |
| --- | --- | --- | --- | --- |
| **Surgical resection (n=111)**  Low WOPS score  High WOPS score | Ref  0.841 | 0.417 | 1.697 | 0.628 |
| **Palliative systemic treatment (n=138)**  Low WOPS score  High WOPS score | Ref  1.162 | 0.727 | 1.857 | 0.530 |
| **Best supportive care (n=66)**  Low WOPS score  High WOPS score | Ref  1.437 | 0.721 | 2.865 | 0.303 |

**Supplementary figure 1. Kaplan Meier curves displaying overall survival in patients who received surgical resection, palliative systemic treatment and BSC**

*Abbreviation: OS=overall survival*
